# Oxidative Damage and Antioxidant Defense in *Sesamum indicum* after Different Waterlogging Durations

**DOI:** 10.3390/plants8070196

**Published:** 2019-06-29

**Authors:** Taufika Islam Anee, Kamrun Nahar, Anisur Rahman, Jubayer Al Mahmud, Tasnim Farha Bhuiyan, Mazhar Ul Alam, Masayuki Fujita, Mirza Hasanuzzaman

**Affiliations:** 1 Department of Agronomy, Faculty of Agriculture, Sher-e-Bangla Agricultural University, Sher-e-Bangla Nagar, Dhaka 1207, Bangladesh; 2 Department of Agricultural Botany, Faculty of Agriculture, Sher-e-Bangla Agricultural University, Sher-e-Bangla Nagar, Dhaka 1207, Bangladesh; 3 Department of Agroforestry and Environmental Sciences, Faculty of Agriculture, Sher-e-Bangla Agricultural University, Sher-e-Bangla Nagar, Dhaka 1207, Bangladesh; 4 Institute of Seed Technology, Sher-e-Bangla Agricultural University, Sher-e-Bangla Nagar, Dhaka 1207, Bangladesh; 5 Laboratory of Plant Stress Responses, Department of Applied Biological Science, Faculty of Agriculture, Kagawa University, Miki-cho, Kita-gun, Kagawa 761-0795, Japan

**Keywords:** flooding, reactive oxygen species, antioxidants, glyoxalase system

## Abstract

The present study was designed to investigate the duration-dependent changes in the biochemical attributes of sesame in response to waterlogging stress. Sesame plants (*Sesamum indicum* L. cv. BARI Til-4) were subjected to waterlogging for 2, 4, 6, and 8 days during the vegetative stage and data were measured following waterlogging treatment. The present study proves that waterlogging causes severe damage to different attributes of the sesame plant. The plants showed an increasing trend in lipid peroxidation as well as hydrogen peroxide (H_2_O_2_) and methylglyoxal contents that corresponded to increased stress duration. A prolonged period of waterlogging decreased leaf relative water content and proline content. Photosynthetic pigments, like chlorophyll (chl) *a*, *b*, and chl (*a*+*b*) and carotenoid contents, also decreased over time in stressed plants. Glutathione (GSH) and oxidized glutathione (GSSG) contents increased under waterlogging, while the GSH/GSSG ratio and ascorbate content decreased, indicating the disruption of redox balance in the cell. Ascorbate peroxidase, monodehydroascorbate reductase, and glutathione peroxidase activity increased under waterlogging, while dehydroascorbate reductase, glutathione reductase, and catalase activity mostly decreased. Waterlogging modulated the glyoxalase system mostly by enhancing glyoxalase II activity, with a slight increase in glyoxalase I activity. The present study also demonstrates the induction of oxidative stress via waterlogging in sesame plants and that stress levels increase with increased waterlogging duration.

## 1. Introduction

Due to the erratic pattern of rainfall and other extreme climate events, waterlogging (or flooding) has now become a major threat to crop production worldwide. Anthropogenic causes, like improper drainage practices, dam failures, and soil erosion, are also sometimes responsible for unwanted flooding [1,2]. Like other environmental stresses (e.g., drought, salinity, heavy metals, extreme temperatures, etc.), the effects of waterlogging stress are intricate, as the magnitude of stress varies not only with plant species, but also with temperature, soil condition, plant age, humidity, sunlight, evaporation, stress duration, water level, etc. [3,4,5]. Waterlogging results in various morphological, physiological, and anatomical changes in plants that are mostly harmful to plant growth and development. For example, waterlogging causes leaf senescence, chlorosis, necrosis, wilting, stunted growth, flower abortion, delayed maturity, and fruit drop, as well as makes plants prone to disease and pest infestation. Waterlogging decreases plant-available oxygen (O_2_; [6]). In plants, hypoxic conditions (deficiency of O_2_) are created as a consequence of short duration waterlogging or flooding of soil and, subsequently, plant roots are exposed to anoxic conditions (absence of O_2_) when waterlogged for a longer period [7]. Lack of O_2_ hampers plant root respiration, which in turn notably inhibits the energy status of root cells. Both the Krebs cycle and the electron transport system become blocked due to O_2_ unavailability, as O_2_ is the terminal electron acceptor of aerobic respiration. Alternatively, waterlogged plants use fermentative metabolism for adenosine triphosphate (ATP) production, although only two ATP are produced per glucose molecule in the fermentation pathway, whereas in aerobic respiration the number of ATP produced is 36. In addition, O_2_ deficit conditions hamper root permeability and also cause root injury, which decreases hydraulic conductivity [8,9]. Consequently, a significant reduction in the net photosynthetic rate and in transpiration is observed due to stomata closure [10,11]. However, such a decline in photosynthetic activity may also be caused by decreased chlorophyll (chl) content, decreased leaf area, and leaf senescence [12]. In addition, waterlogging-induced injury to roots leads to some biochemical alterations e.g., hindered ribulose bisphosphate carboxylase (RuBPC), glycolate oxidase, and phosphoglycolate activity; destruction of chloroplast membranes, which result in decreased efficiency of photosynthetic electron transport and photosystem II [11]. Such photosynthesis impairment in plants under waterlogging stress augments the production of reactive oxygen species (ROS), such as superoxide (O_2_^•−^), singlet oxygen (^1^O_2_), hydrogen peroxide (H_2_O_2_), and hydroxyl radicals (OH^•^) to a level that is harmful for plants [13]. Different cellular organelles (mitochondria, chloroplast, peroxisomes, etc.) are known to be the sites of ROS generation [14]. Together with altered physiological and growth processes, waterlogging severely affects reproductive development, resulting in reduced yields, as reported in a number of plant species [15,16,17,18].

To cope with the morphological, physiological, cellular, or oxidative damage caused by waterlogging stress, the plant itself shows some protective or adaptive mechanisms. Adventitious root formation and stem elongation are examples of morphological adaptations, while aerenchyma formation is the most common type of anatomical adaptation to waterlogging stress. Switching to anaerobic fermentation, through use of the alcohol dehydrogenase enzyme, is a method of biochemical adaptation. The most important adaptive mechanism, however, is the well-balanced antioxidant defense system that facilitates scavenging of the harmful ROS, consisting of both enzymatic, e.g., catalase (CAT); glutathione peroxidase (GPX); ascorbate peroxidase (APX); monodehydroascorbate reductase (MDHAR); dehydroascorbate reductase (DHAR); and glutathione reductase (GR) and non-enzymatic components, e.g., ascorbate (AsA); glutathione (GSH); tocopherols; and carotenoids [1,19]. The activity of the components in this system varies greatly with stress duration and plant genotype [5].

Methylglyoxal (MG) is another toxic compound produced spontaneously under both biotic and abiotic stress conditions in the glycolysis pathway. However, cytotoxic MG can be detoxified by the glyoxalase system through a two-step reaction; each step includes an enzyme. The first enzyme, glyoxalase I (Gly I), uses GSH to convert MG into *S*-D-lactoylglutathione (SLG). The second enzyme, glyoxalase II (Gly II), converts SLG into D-lactic acid while restoring GSH. This demonstrates the importance of endogenous GSH availability for the glyoxalase system [20]. The coordinated action of the antioxidant defense and glyoxalase systems helps plants persist under environmental or abiotic stress conditions by inducing a tolerance mechanism [21].

Sesame is extremely susceptible to waterlogging and continuous heavy rains. When grown on soils with poor drainage, sesame is adversely affected and can suffer yield losses of more than 30% (in severe cases, 50–90%) [22]. Field experiments on waterlogged sesame have recorded premature senescence resulting from leaf chlorosis, necrosis, defoliation, and reduced nitrogen fixation, leading to the cessation of growth and reduced yields [23]. Waterlogging-induced oxidative stress and antioxidant enzyme activity, along with the formation of distinct aerenchyma, have also been reported in sesame [24]. A number of studies demonstrate the morphophysiological, anatomical, and biochemical responses of sesame under flooding stress [22,23,24], but sesame’s oxidative stress responses are yet to be explored. Our experiment was aimed at understanding the coordinated actions of antioxidant defense and glyoxalase systems in conferring waterlogging stress tolerance in sesame. BARI Til-4 is a popular variety and often experience heavy rain and subsequent waterlogging. Therefore, we aimed at investigating the plant responses to this stress.

## 2. Materials and Methods

### 2.1. Plant Materials and Stress Treatments

Sesame (*Sesamum indicum* L. cv. BARI Til-4) seeds were sown in plastic pots (12 kg of soil per pot) and placed in an experimental shed. The moisture content of the soil was 60% field capacity and shed humidity was 75–80% during the experimental procedure. At 21 days after sowing, plants were exposed to waterlogging treatment. The waterlogged condition was created by saturating the soil with sufficient water to have at least 2 cm of standing water on the soil surface and the opening of the pot was closed tightly to prevent water loss. Plants remained in waterlogged conditions for specific durations (2, 4, 6, and 8 days), following which the water was drained from the pots and leaves were harvested for data measurement. The experiment followed completely randomized design (CRD) procedures and was replicated three times. The experiment undertook four treatments and used different control plants for each treatment, to maintain comparable plant age.

### 2.2. Relative Water Content Measurement

Barrs and Weatherly’s [25] method was followed to measure the relative water content (RWC) of sesame leaves. Fresh leaves were weighed (fresh weight, FW) and then kept in Petri plates making those float on enough water up to 8 h. Then the extra water from the leaf surface was soaked and weighed again (turgid weight, TW). For the next 48 h, those leaves were dried in the oven, and the final weight (dry weight, DW) was measured. Following is the formula used to calculate the leaf RWC:

$$\mathrm{RWC}\;(\%)=\frac{FW-DW}{TW-DW}\times 100$$

### 2.3. Photosynthetic Pigments Measurement

The method described by Arnon [26] was followed to quantify the photosynthetic pigments in leaves. 80% (*v*/*v*) acetone (10 mL) was used to extract 0.5 g of fresh leaves and the extract was then centrifuged at 2000× *g* for 10 min. The obtained supernatant was diluted and the absorbance was measured with the help of a UV-visible spectrophotometer. Three different wavelengths were selected for measuring chl *a*, chl *b* and carotenoid (Car) contents which were 663, 645 and 480 nm, respectively.

### 2.4. Measurement of Proline Content

The content of proline (Pro) in sesame leaves was measured following the procedure of Bates et al. [27]. Leaves (0.5 g) were extracted in 3% sulfosalicylic acid (5 mL) while maintaining ice-cold conditions in a mortar-pestle. Then, the extracted homogenate was centrifuged at 11,500× *g* for 15 min. One mL of supernatant was taken from each sample and mixed with acidic ninhydrin (1 mL) and then with glacial acetic acid (1 mL). The mixture was then kept for 1 h in a hot (100 °C) water bath and then kept on ice for cooling after transferring to test tubes. Then toluene (2 mL) was added to the cold mixture, and vortexed to mix it well. The Pro content of the sample was determined by comparing with a standard curve of known concentration of Pro.

### 2.5. Measurement of Lipid Peroxidation

The content of malondialdehyde (MDA) as a marker of lipid peroxidation, was measured following Heath and Packer’s [28] method with slight modifications from Hasanuzzaman et al. [29]. Trichloroacetic acid (TCA, 5% (w/v), 3 mL) was used to homogenize 0.5 g of leaves. Then homogenized samples were centrifuged at 11,500× *g* for 15 min. Thiobarbituric acid (TBA) reagent (0.5% of TBA in 20% TCA) was used as reaction buffer, 4 mL of which was added to 1 mL of leaf supernatant and the mixture was heated in a hot water bath at 95 °C. After 30 min of heating, the mixture was then cooled quickly in an ice bath for 10 min. The absorbance was then measured at 532 nm and 600 nm, later one for correction of non-specific absorbance. Malondialdehyde content was calculated by using extinction coefficient 155 mM^−1^ cm^−1^ and expressed as nmol g^−1^ FW.

### 2.6. Determination of Hydrogen Peroxide Content

The procedure described by Yu et al. [30] was followed to measure H_2_O_2_ content. Leaf samples (0.5 g) were homogenized in 3 mL of potassium–phosphate (K–P) buffer (50 mM; pH 6.5) and then centrifuged for 15 min at 11,500× *g*. TiCl_4_ (666.4 μL, 0.1%) in 20% H_2_SO_4_ (*v*/*v*) was then added to 2 mL of supernatant and the mixture kept at room temperature for 10 min before centrifuging again at 11,500× *g* for 12 min. The spectrophotometer wavelength was set at 410 nm and the absorbance was then measured. The extinction coefficient was 0.28 μM^−1^ cm^−1^, which was used to quantify the H_2_O_2_ content.

### 2.7. Measurement of Methylglyoxal Level

The content of MG was obtained following the procedure of Wild et al. [31]. Perchloric acid (5%) was used as extraction buffer and the mixture was then centrifuged for 10 min at 11,000× *g*. To remove the color of the supernatant charcoal was added and then the colorless supernatant was added to saturated sodium carbonate solution to undergo neutralization. For the estimation of MG, the neutralized supernatant was added to sodium dihydrogen phosphate and n-acetyl-l-cysteine to make 1 mL of the final volume. After 10 min, the formation of n-α-acetyl-*S*-(1-hydroxy- 2-oxoprop-1-yl) cysteine was measured at 288 nm, and the MG content was calculated using a standard curve of known concentration.

### 2.8. Extraction and Measurement of Ascorbate and Glutathione

Leaves (0.5 g) were homogenized in ice-cold 5% metaphosphoric acid (3 mL) which contained 1 mM ethylenediaminetetraacetic acid (EDTA) and then centrifuged at 11,500× *g* for 12 min at 4 °C. The collected supernatant was used to analyze AsA and GSH according to the procedure described by Huang et al. [32]. After neutralizing the supernatant with 0.5 M K-P buffer (pH 7.0), 0.1 M of dithiothreitol was used for the reduction of the oxidized fraction. Ascorbate was assayed by recording the changes of absorbance spectrophotometrically at 265 nm in 100 mM K-P buffer (pH 7.0) with 0.5 units of ascorbate oxidase (AO). A specific standard curve of AsA was used for quantification. For the measurement of GSH pool, method of Yu et al. [30] was followed with slight modifications [33]. Supernatants (0.2 mL) were added with 0.5 M K-P buffer (0.3 mL) for neutralization, and then GSH was oxidized by 5,5-dithio-bis(2-nitrobenzoic acid) (DTNB) and reduced by nicotinamide adenine dinucleotide phosphate (NADPH) in the presence of GR. Then at 412 nm wavelength in a spectrophotometer, the absorbance was measured to calculate GSH content. This GSH content evaluates the rate of absorption changes during the generation of 2-nitro- thiobenzoic acid (NTB) from the reduction of DTNB. Oxidized glutathione (GSSG) was determined after removing GSH by 2-vinylpyridine derivatization. Two standard curves were made using known concentrations of GSH and GSSG and used for the calculation. The content of GSH was calculated by subtracting GSSG from total GSH.

### 2.9. Enzyme Extraction and Assays

A mixture of ice-cold K-P buffer (50 mM, pH 7.0) containing 100 mM KCl, 1 mM AsA, 5 mM β-mercaptoethanol, and 10% (w/v) glycerol was used as the extraction buffer for enzyme assays. Leave samples (0.5 g) were homogenized using a pre-cooled mortar and pestle adding 1 mL of the abovementioned extraction buffer. Then the mixture was centrifuged taking in Eppendorf tubes for 10 min at a speed of 11,500× *g*. The supernatants were used for the measurement of enzymatic activity.

This experiments to assay the CAT (EC: 1.11.1.6) activity followed the method explained by Hasanuzzaman et al. [29]. The reaction mixture was prepared with 50 mM K-P buffer (pH 7.0), 15 mM H_2_O_2_, and enzyme solution making a final volume of 700 μL and the decrease of absorbance was recorded at 240 nm for 1 min. The extinction coefficient used for calculation was 39.4 mM^−1^ cm^−1^.

Nakano and Asada [34] described the procedure of APX (EC: 1.11.1.11) activity measurement which was followed. The reaction mixture was prepared with 50 mM K-P buffer (pH 7.0), 0.5 mM AsA, 0.1 mM H_2_O_2_, 0.1 mM EDTA, and enzyme extract making a final volume of 700 μL and the decrease in absorbance was observed at 290 nm for 1 min. The extinction coefficient used for calculation was 2.8 mM^−1^ cm^−1^.

Here the methodology of Hossain et al. [35] was used to quantify MDHAR (EC: 1.6.5.4) activity. 50 mM Tris-HCl buffer (pH 7.5), 0.2 mM NADPH, 2.5 mM AsA, 0.5 units of ascorbate oxidase (AO), and enzyme extract making a final volume of 700 μL was used as the reaction solution and the change in absorbance was measured at 340 nm for 1 min. The extinction coefficient used for calculation was 6.2 mM^−1^ cm^−1^.

Like APX, the Nakano and Asada [34] suggested method of DHAR (EC: 1.8.5.1) activity measurement was also used. Reaction mixtures were prepared with 50 mM K-P buffer (pH 7.0), 2.5 mM GSH, 0.1 mM EDTA, 0.1 mM dehydroascorbate (DHA) and enzyme extract. Then the change in absorbance was recorded at 265 nm for 1 min. The extinction coefficient used for calculation was 14 mM^−1^ cm^−1^.

The activity of GR (EC: 1.6.4.2) was assayed according to the procedure followed by Hasanuzzaman et al. [29]. 0.1 M K-P buffer (pH 7.0), 1 mM EDTA, 1 mM GSSG, 0.2 mM NADPH, and enzyme extract all together served the purpose of the reaction mixture and the decrease in absorbance was monitored at 340 nm for 1 min. The activity was calculated using an extinction coefficient of 6.2 mM^−1^ cm^−1^.

Elia et al. [36] described the method of assaying GPX (EC: 1.11.1.9) activity which was followed by our experiments. Preparation of the reaction solution was done with 100 mM K-P buffer (pH 7.0), 1 mM EDTA, 1 mM sodium azide (NaN_3_), 0.12 mM NADPH, 2 mM GSH, 1 unit GR, 0.6 mM H_2_O_2_ (as a substrate), and 20 μL of sample solution. The change in absorbance was monitored for 1 min at 340 nm, and the extinction coefficient used for calculation was 6.62 mM^−1^ cm^−1^.

The first enzyme of the glyoxalase system Gly I (EC: 4.4.1.5) activity was measured according to the procedure explained in Hasanuzzaman et al. [29]. The reaction solution was prepared with 100 mM K–P buffer (pH 7.0), 15 mM magnesium sulfate, 1.7 mM GSH, and 3.5 mM MG in a final volume of 700 μL. The increase in absorbance was recorded at 240 nm for 1 min. The extinction coefficient used for calculation was 3.37 mM^−1^ cm^−1^.

To assay the activity of the second enzyme Gly II (EC: 3.1.2.6) the procedure described by Principato et al. [37] was followed. The components of the reaction mixture were 100 mM Tris-HCl buffer (pH 7.2), 0.2 mM DTNB, and 1 mM SLG and plant sample. The change in absorbance was recorded at 412 nm for 1 min. The extinction coefficient used for calculation was 13.6 mM^−1^ cm^−1^.

### 2.10. Statistical Analysis

The experiments followed a CRD design and three replicates were run. All data measured were subjected to analysis of variance (ANOVA). The mean differences were compared by Duncan’s multiple range test (DMRT) and correlation analysis was done using XLSTAT v.2018 software (AddinSoft, New York, USA). Differences with *p* ≤ 0.05 were considered significant.

## 3. Results

### 3.1. Leaf Relative Water Content

**Figure 1:**
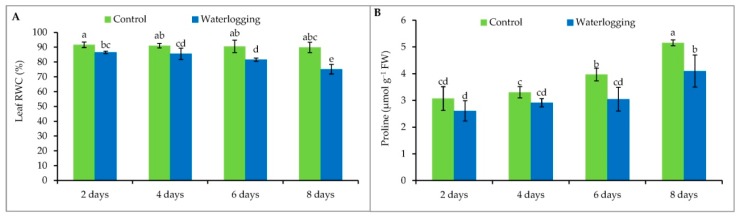
Changes in RWC (**A**) and Pro (**B**) content of leaves from sesame plants waterlogged during the vegetative stage. Mean (±SD) was calculated based on three replications of each treatment. Values in a bar with different letters are significantly different at *p* ≤ 0.05 applying LSD test.

Leaf wilting symptoms were observed in plants under waterlogging stress (Supplementary Figure S1) because of the reduction in leaf RWC. This study showed a significant reduction of leaf RWC in all the plants that were waterlogged during their vegetative stage, compared to their respective controls. The smallest reduction in RWC was observed in plants waterlogged for 2 days; RWC continued to decrease with increased duration of waterlogging. Plants waterlogged for up to 8 days showed the lowest RWC (75%), while their control had 90% leaf RWC (Figure 1A).

### 3.2. Proline Content

Compared to control, Pro content remained unchanged after waterlogging for 2 and 4 days but it decreased at 6th and 8th day of waterlogging which was 23 and 20% lower than control (Figure 1B).

### 3.3. Chlorophyll Content

**Figure 2:**
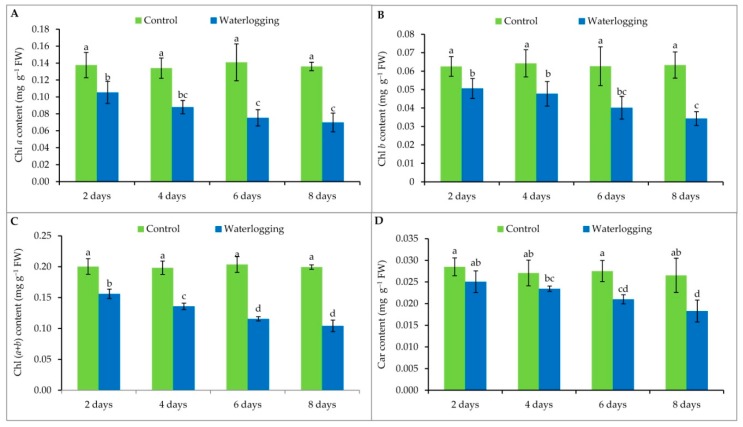
Contents of chl *a* (**A**), chl *b* (**B**), chl (*a* + *b*) (**C**) and carotenoids (**D**) of sesame leaves from plants affected by waterlogging stress at vegetative stage. Mean (±SD) was calculated from three replicates for each treatment. Values in a bar with different letters are significantly different at *p* ≤ 0.05 applying LSD test.

Chlorophyll content of sesame leaves was greatly reduced with increasing duration of waterlogging (Figure 2). The lowest chl *a* content was recorded in the plants waterlogged for 8 days. The same trend was observed for chl (*a* + *b*) and for carotenoid contents. Chlorophyll *b* content was also lower in all waterlogged plants compared to their respective controls (Figure 2).

### 3.4. Lipid Peroxidation and Hydrogen Peroxide Content

**Figure 3:**
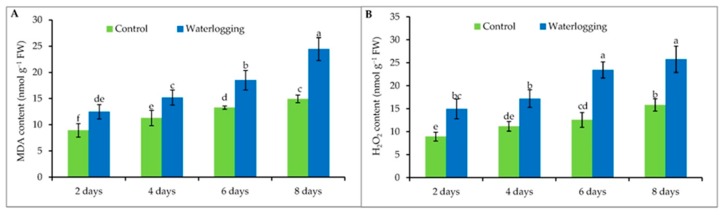
(**A**) MDA and (**B**) H_2_O_2_ contents of sesame leaves from plants affected by waterlogging stress at vegetative stage. Mean (±SD) was calculated from three replicates for each treatment. Values in a column with different letters are significantly different at *p* ≤ 0.05 applying LSD test.

Waterlogging stress resulted in a significant increase in MDA content, compared with the controls. The level of MDA production increased in a time-dependent manner. The highest MDA levels were recorded in plants waterlogged for 8 days which was 39% higher than the control. Similar results were obtained for the plants waterlogged for 6 days (Figure 3A).

A sharp increase of H_2_O_2_ was observed when seedlings were exposed to the waterlogged condition. Like lipid peroxidation levels, plants waterlogged for 8 days showed the highest H_2_O_2_ content, which was similar to that of plants that were waterlogged for 6 days. Also, the H_2_O_2_ content of plants waterlogged for 4 days was similar to that of plants waterlogged for 2 days (Figure 3B).

### 3.5. Ascorbate and Glutathione Contents

**Figure 4:**
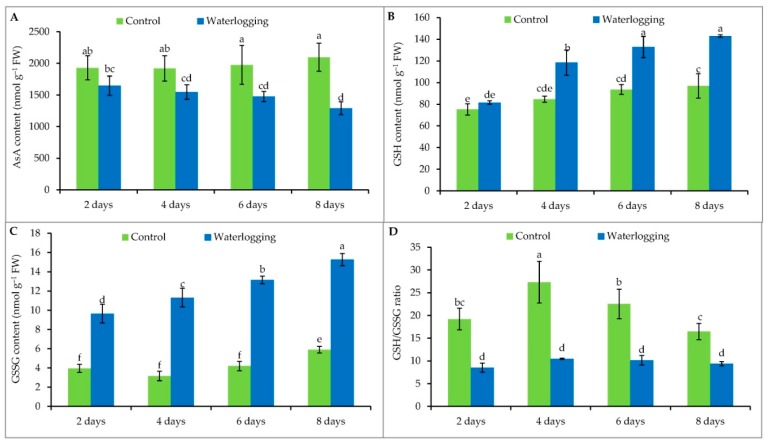
AsA (**A**), GSH (**B**), GSSG (**C**) contents and GSH/GSSG (**D**) ratio of sesame leaves from plants affected by waterlogged condition for different durations at vegetative stage. Mean (±SD) was calculated from three replicates for each treatment. Values in a bar with different letters are significantly different at *p* ≤ 0.05 applying LSD test.

The AsA content in sesame leaves decreased under waterlogging stress, compared to the controls. After 2, 4, 6, and 8 days of the waterlogged condition, AsA content was 15%, 19%, 25%, and 38% lower compared to levels in the control plants, respectively (Figure 4A).

Glutathione content increased in the stressed plants when they were waterlogged for 4 or more days, compared to the controls (Figure 4B). GSSG levels were higher in all of the waterlogged plants compared to their controls (Figure 4C). However, changing the duration of waterlogging did not have a significant effect on the GSH/GSSG ratio of sesame plants (Figure 4D).

### 3.6. Antioxidant Enzyme Activity

**Figure 5:**
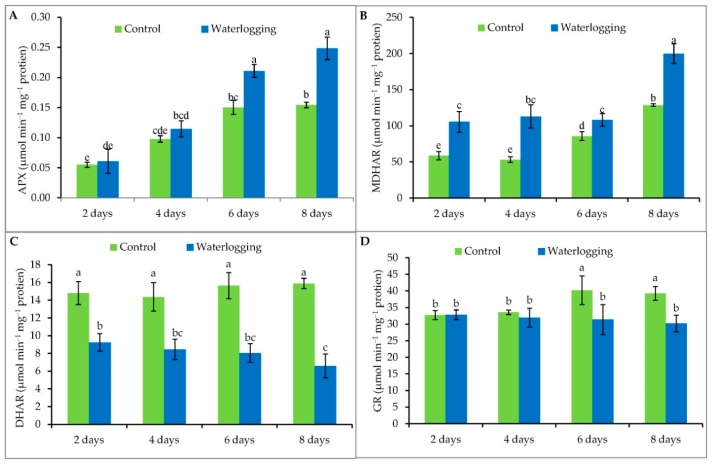
The activity of APX (**A**), MDHAR (**B**), DHAR (**C**) and GR (**D**) enzyme in sesame leaves from plants affected by waterlogged condition for different durations at vegetative stage. Mean (±SD) was calculated from three replicates for each treatment. Values in a bar with different letters are significantly different at *p* ≤ 0.05 applying LSD test.

Waterlogging stress increased APX activity by 10%, 17%, 40%, and 61% in plants waterlogged for 2, 4, 6, and 8 days, respectively, compared to their controls (Figure 5A). Higher MDHAR activity was observed under waterlogging stress compared to same-age well-drained plants. Unlike APX, however, there was no time-dependent increase in MDHAR activity. Rather, MDHAR levels suddenly increased by 55% at 8 days of waterlogging, compared to the control plants (Figure 5B). Our experiment also showed a significant reduction in DHAR activity by 38%, 42%, 49%, and 59% in plants waterlogged for 2, 4, 6, and 8 days, respectively, compared to same-age well-drained plants. However, the highest reduction occurred in plants waterlogged for the longest duration (8 days) (Figure 5C). Reduced GR enzyme activity levels were recorded when plants were waterlogged for 6 (22%) or 8 (23%) days, whereas GR enzyme activity levels were not affected in plants waterlogged for up to 4 days, compared to the controls (Figure 5D).

**Figure 6:**
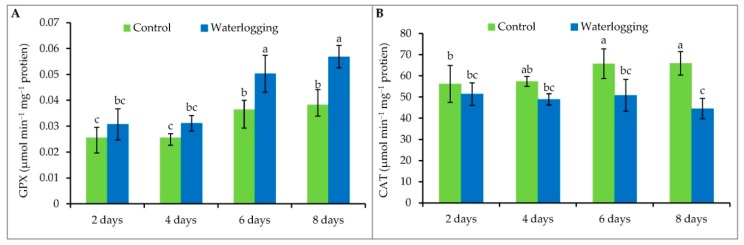
The activity of GPX (**A**) and CAT (**B**) enzymes in sesame leaves from plants affected by waterlogged condition for different durations at vegetative stage. Mean (±SD) was calculated from three replicates for each treatment. Values in a bar with different letters are significantly different at *p* ≤ 0.05 applying LSD test.

Plants waterlogged for 6 days and 8 days both showed significantly higher (38% and 47%, respectively) GPX activity compared to their controls (Figure 6A). A gradual decline in CAT enzyme activity levels (9%, 10%, 23%, and 33% at 2, 4, 6, and 8 days of waterlogging, respectively) were observed compared to the control plants (Figure 6B).

Total protein content of plants under different durations of waterlogging is reported in Supplementary Figure S2.

### 3.7. Methylglyoxal Detoxification

**Figure 7:**
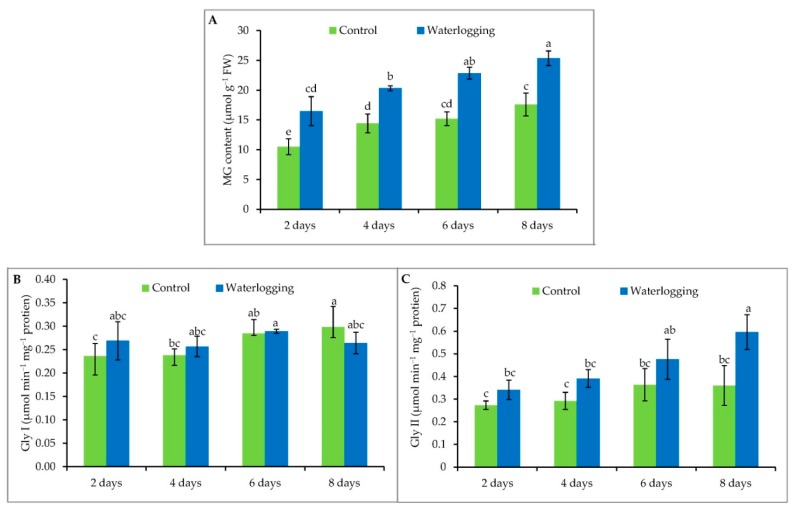
MG content (**A**), Gly I (**B**) and Gly II (**C**) activities in sesame leaves from plants affected by waterlogged condition for different durations at vegetative stage. Mean (±SD) was calculated from three replicates for each treatment. Values in a bar with different letters are significantly different at *p* ≤ 0.05 applying LSD test.

Upon exposure to waterlogging conditions, MG content increased significantly compared to controls. A 60%, 42%, 46%, and 47% increase in MG content was observed in sesame seedlings waterlogged for 2, 4, 6, and 8 days, respectively, compared to their respective control plants (Figure 7A). On the other hand, the two related enzymes—Gly I and Gly II showed a divergent mode of activity under the same conditions. The activity of Gly I was higher at 2 days of waterlogging, declined slightly at 4 days, with a subsequent increase at 6 days of waterlogging (Figure 7B). However, Gly I again decreased sharply at 8 days of waterlogging, which may be due to excessive production of cytotoxic MG and lower production of GSH (Figure 7B). Unlike Gly I, Gly II activity was enhanced with increasing duration of waterlogging, with the greatest activity in the plants waterlogged for 8 days (Figure 7C).

### 3.8. Correlation among the Parameters

**Figure 8:**
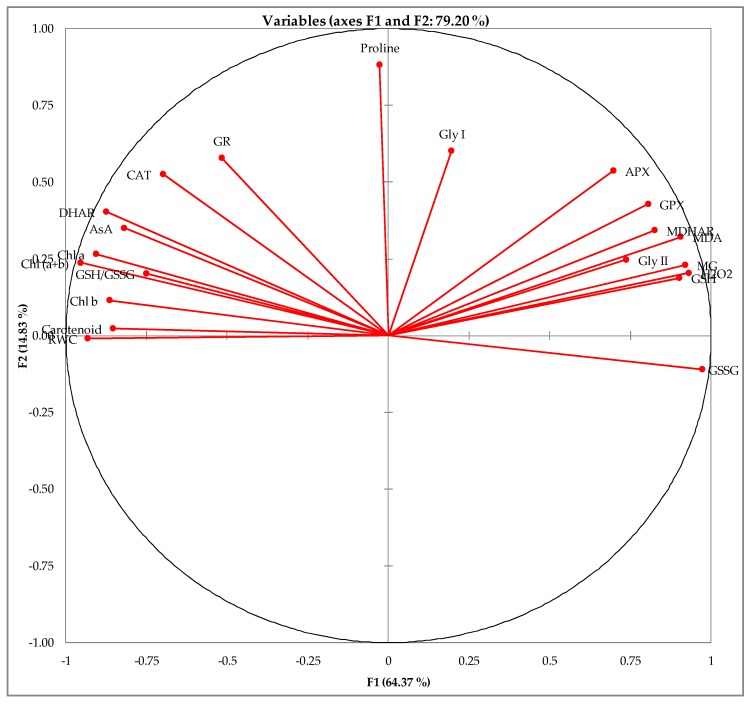
Principal component analysis (PCA) of different studied attributes.

From the correlation study, it is clear that oxidative stress markers such as H_2_O_2_ and MDA were negatively correlated (*p* ≤ 0.05) with most of the antioxidant enzymes as well as non-enzymatic antioxidants (i.e., AsA, GSH) and their redox state (Table 1). The similarities between the different studied attributes are presented in Figure 8. The components of the antioxidant defense and glyoxalase systems are highly affected by the treatments; the exceptions were chl *a*, GR and Gly I which were not substantially affected (Figure 8).

## 4. Discussion

A decrease in leaf RWC denotes a limitation of water availability for cell expansion [38]. In spite of the excess amount of water available under flooded or waterlogged conditions, sesame plants showed reduced leaf RWC. This could be due to a prevalence of hypoxic or anoxic conditions which hampered root permeability [11] and, as a result, wilted leaves were observed on sesame seedlings. Similar reductions in leaf RWC due to waterlogging stress were also reported in pineapple [39] and mung bean [18]. Proline acts as an osmoprotectant molecule, maintaining and improving the water status of plants. Proline also acts as an antioxidant, protecting the cell from free radical damage and maintaining the cellular environment for the better synthesis of biomolecules that play a role in stress adaptation [40]. Unlike many other studies, Pro content was decreased in plants grown under prolonged waterlogging (6 and 8 days), which was supported by other studies [41,42,43,44]. This may be due to the lower tolerance or higher susceptibility of this particular sesame cultivar, leading to the reduced osmotic adjustment capacity of plant cells.

Under waterlogging stress, leaf yellowing might occur due to a reduction in leaf nitrogen [45], nodulation, and N fixation. Production of toxic substances, such as nitrites and sulfides, which move from the soil through roots to the leaves (if carried upward in large quantities), may occur [15]. In addition, waterlogging results in reduced soil nitrogen through rapid volatilization and denitrification [17]. In the present study, a time-dependent reduction of leaf photosynthetic pigments was observed. Consistently, such a reduction of leaf chl due to waterlogging stress was also observed in several crops [18,24,46,47,48,49].

Waterlogging-induced overproduction of ROS and subsequent lipid peroxidation has also been demonstrated in a number of crops, including sesame [24,50,51]. In our study, we also observed a time-dependent enhancement of MDA levels. As the greater production of ROS in waterlogged tissues increases lipid peroxidation, it restricts the antioxidant defense system’s ability to mitigate the stress-generated ROS surplus. Besides MDA, a duration-dependent increase of H_2_O_2_ content was also recorded in our experiment, likely due to the same reason. This excess amount of H_2_O_2_ is noteworthy because it readily permeates membranes and therefore, is not compartmentalized in the cell. H_2_O_2_ may also inactivate enzymes by oxidizing their thiol groups [21]. In the case of both MDA and H_2_O_2_ measurements, a time-dependent increase was recorded in the control plants: as the plants aged, oxidative stress markers accumulated in their chloroplasts [52].

To negate the detrimental effects of ROS, plants are equipped with an array of non-enzymatic scavengers and antioxidant enzymes that act in concert to alleviate cellular damage under oxidative stress conditions. Ascorbate, one of the most abundant non-enzymatic antioxidants, is the substrate of APX, a critical component of the AsA–GSH cycle for H_2_O_2_ detoxification [34,53]. In our present study, AsA content significantly decreased with increasing stress duration, which corroborated other reports under different abiotic stress conditions [54,55]. However, irregular changes in AsA content have also been reported in both tomato and eggplant genotypes that were exposed to waterlogging for 12, 24, 36, 48, 60, and 72 h [56]. Like AsA, GSH also plays a pivotal role in preventing cell oxidative damage by equilibrating redox status. GSH can participate not only in scavenging H_2_O_2_ through the AsA–GSH cycle but also in direct reactions with other ROS [57]. Glutathione content significantly increased in the stressed plants when they were waterlogged for 4 or more days, but no significant difference between the plants waterlogged for 6 and 8 days was observed; probably because of their higher GSSG content, as GSH participates in ROS scavenging. As a result, a notable increase in GSSG content in all the waterlogged plants, compared to their respective controls, was observed. These results for both GSH and GSSG were similar to the results observed in plants exposed to other abiotic stresses, like salinity [54,55], high temperature [58], and drought [59]. When the GSH/GSSG ratio was considered, however, there was no effect on the waterlogged plants; rather, this ratio changed in the control plants themselves based on age and cell signaling mechanisms.

The antioxidant defense system’s enzymatic antioxidants include CAT, APX, MDHAR, DHAR, and GPX, etc. Catalase is the enzyme that actively catalyzes the H_2_O_2_ scavenging reaction. The increasing trend in H_2_O_2_ accumulation consequent to increased stress duration is partly the result of decreased CAT activity as the H_2_O_2_ scavenging enzyme [1]. Glutathione peroxidase functions both as an H_2_O_2_-detoxifying agent and as an oxidative signal transducer [1]. Very few investigations have been documented regarding the effect of waterlogging stress on GPX activity. In this experiment, GPX activity increased in a time-dependent manner with increased duration of waterlogging, which is supported by other studies on sesame [60]. The enzymes of the AsA-GSH cycle (APX, MDHAR, DHAR, and GR) readily and efficiently catalyze ROS detoxification with the help of vital components AsA and GSH; after scavenging ROS, AsA and GSH are recycled [61,62,63]. The APX catalyzes the reduction of H_2_O_2_ to H_2_O by using AsA [64,65]. The univalent oxidation of AsA leads to the formation of MDHA. If MDHA is not reduced again to AsA by MDHAR, it will spontaneously disproportion into AsA and DHA. The regeneration of AsA could be regulated in this cycle by NADPH-dependent MDHAR activity [66]. MDHAR is crucial for AsA regeneration and essential for maintaining a reduced pool of AsA [67]. Dehydroascorbate reductase regenerates AsA from its oxidized state (DHA) and regulates the cellular AsA redox state [1]. Another important enzyme, GR, is responsible for catalyzing the NADPH-dependent reduction of GSSG’s disulfide bond, which eventually maintains the GSH pool [68]. In plant cells, the maintenance of a higher ratio of GSH/GSSG is thus managed by GR, which increases scavenging of H_2_O_2_ under stress conditions [62]. In our study, it was observed that APX and MDHAR activity increased with stress duration, while DHAR and GR activity reduced in a time-dependent manner.

**Figure 9:**
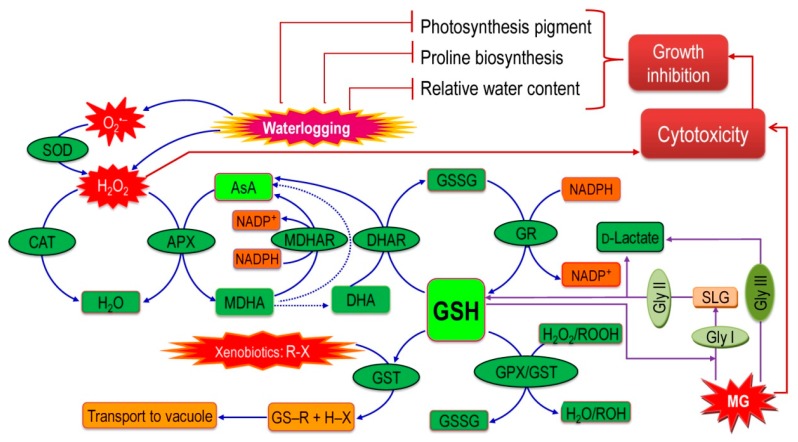
Coordinated interaction of antioxidant defense systems and glyoxalase systems in sesame plants studied under waterlogged condition [20]. R may be an aliphatic, aromatic, or heterocyclic group; X may be a sulfate, nitrite, or halide group. Solid arrows indicate enzymatic reactions while dotted arrows indicate non-enzymatic reactions.

Previously, there were hardly any studies that demonstrated the generation of cytotoxic MG and the activation of glyoxalase system enzymes (Gly I and Gly II) under stress from waterlogging. These findings can, therefore, be considered the paramount contributions of this experiment. A significant increase in MG content was recorded in waterlogged plants, which indicates that the waterlogging-induced oxidative damage is enhanced by MG production in addition to ROS produced. Production of MG also leads to ROS production and elimination of MG similarly reduces ROS toxicity [20]. Our study shows that an increase in GSH content and Gly II activity improves the plant ability to detoxify the increased amount of MG. However, Gly I activity was not boosted in waterlogged sesame in the longer period even if a slight increase was observed after 2 days, indicating that Gly I is possibly activated at the initial stage of waterlogging, diminishing as the plants become severely damaged upon a prolonged stress. Importantly, GSH play a central role in both of the systems because GSH produced by AsA-GSH system is used by the glyoxalase system, which is again regenerated. Therefore, their good coordination is vital in detoxifying both ROS and MG ([69] Figure 9).

## 5. Conclusions

Global climate change is leading to the unpredictable occurrence of heavy rainfall and an increase in atmospheric temperatures, ultimately resulting in sea-level rises. As a result, crops are facing recurrent episodes of localized rainfall and flooding conditions, which may lead to a dramatic decreases in the world’s crop production, so it is high time to combine new research with existing knowledge about crops’ tolerance to abiotic stresses in order to invent new crop varieties that are resistant to waterlogging. Alternatively, promising agronomic approaches that could help farmers avoid such damage in the first place should also be explored. This study found that the impacts of waterlogging stress on sesame plants depend mainly on stress duration, irrespective of the stage of growth. Waterlogging-induced oxidative stress was prominent, as indicated by the remarkable increase in MDA and H_2_O_2._ However, the antioxidant defense system, which can protect plants from this oxidative damage, is comprised of some non-enzymatic and enzymatic antioxidants, which were studied. Results showed a waterlogging-induced reduction in AsA content as well as an enhancement of GSH content, which has also been found in response to other abiotic stresses. APX, MDHAR, and GPX activity increased alongside increased stress duration and CAT, DHAR, and GR activity was reduced in a time-dependent manner. Importantly, coordinated interaction of the antioxidant defense system and glyoxalase systems played a major role in detoxifying ROS and MG, which protects plants from oxidative stress and cellular damage (Figure 9). This promising phenomenon warrants further investigation at the gene level in order to discover its underlying mechanisms and tailoring the traits associated with ROS detoxification and MG detoxification systems. Determining the cause of sesame’s current limited adaptability may further assist in pinpointing genes that are susceptible or tolerant to waterlogging and eventually lead to the production of tolerant varieties.

## Figures and Tables

**Table 1:** Correlation matrix of different observed parameters activities in sesame leaves from plants affected by waterlogged condition for different durations at vegetative stage.

Variables	RWC	Pro	Chl *a*	Chl *b*	Chl (*a* + *b*)	Car	MDA	H_2_O_2_	AsA	GSH	GSSG	GSH/GSSG	APX	MDHAR	DHAR	GR	GPX	CAT	MG	Gly I	Gly II
RWC	**1**	−0.035	**0.808**	**0.808**	**0.862**	**0.790**	**−0.844**	**−0.816**	**0.811**	**−0.832**	**−0.876**	**0.631**	**−0.656**	**−0.804**	**0.781**	**0.496**	**−0.793**	**0.708**	**−0.833**	−0.138	**−0.665**
Proline		**1**	0.258	0.181	0.252	0.012	0.253	0.168	0.298	0.129	−0.147	0.210	**0.452**	0.373	0.395	**0.408**	0.325	**0.443**	0.128	0.349	0.140
Chl *a*			**1**	**0.705**	**0.975**	**0.847**	**−0.710**	**−0.813**	**0.862**	**−0.805**	**−0.918**	**0.742**	**−0.490**	**−0.618**	**0.897**	**0.573**	**−0.621**	**0.733**	**−0.752**	−0.052	**−0.529**
Chl *b*				**1**	**0.844**	**0.585**	**−0.741**	**−0.784**	**0.677**	**−0.721**	**−0.862**	**0.688**	**−0.570**	**−0.671**	**0.849**	**0.513**	**−0.669**	**0.624**	**−0.803**	−0.085	**−0.632**
Chl (*a* + *b*)					**1**	**0.823**	**−0.767**	**−0.859**	**0.863**	**−0.833**	**−0.962**	**0.775**	**−0.548**	**−0.676**	**0.943**	**0.593**	**−0.678**	**0.749**	**−0.818**	−0.066	**−0.596**
Car						**1**	**−0.741**	**−0.794**	**0.777**	**−0.790**	**−0.843**	**0.638**	**−0.607**	**−0.709**	**0.710**	**0.425**	**−0.629**	**0.535**	**−0.725**	−0.092	**−0.566**
MDA							**1**	**0.900**	**−0.597**	**0.871**	**0.822**	**−0.556**	**0.808**	**0.879**	**−0.662**	−0.307	**0.859**	**−0.501**	**0.912**	0.291	**0.837**
H_2_O_2_								**1**	**−0.634**	**0.877**	**0.889**	**−0.679**	**0.757**	**0.801**	**−0.759**	−0.364	**0.817**	**−0.512**	**0.897**	0.279	**0.752**
AsA									**1**	**−0.732**	**−0.814**	**0.562**	**−0.418**	**−0.545**	**0.830**	**0.627**	**−0.543**	**0.736**	**−0.632**	0.034	**−0.422**
GSH										**1**	**0.840**	**−0.519**	**0.765**	**0.740**	**−0.691**	−0.291	**0.817**	**−0.538**	**0.891**	0.213	**0.666**
GSSG											**1**	**−0.861**	**0.600**	**0.780**	**−0.915**	**−0.530**	**0.706**	**−0.685**	**0.878**	0.194	**0.646**
GSH/GSSG												**1**	−0.300	**−0.650**	**0.793**	**0.414**	**−0.461**	**0.537**	**−0.639**	−0.182	**−0.438**
APX													**1**	**0.658**	−0.377	−0.195	**0.835**	−0.102	**0.778**	0.359	**0.511**
MDHAR														**1**	**−0.586**	−0.194	**0.748**	**−0.434**	**0.808**	0.285	**0.709**
DHAR															**1**	**0.630**	**−0.509**	**0.767**	**−0.720**	0.054	**−0.531**
GR																**1**	−0.153	**0.674**	−0.345	0.226	−0.221
GPX																	**1**	−0.351	**0.817**	0.359	**0.661**
CAT																		**1**	**−0.539**	0.190	**−0.565**
MG																			**1**	0.369	**0.718**
Gly I																				**1**	0.320
Gly II																					**1**

Values in bold are different from 0 with a significance level alpha = 0.05.

## Supplementary Materials

The following are available online at https://www.mdpi.com/2223-7747/8/7/196/s1, Figure S1: Phenotypic appearance of sesame plants under different levels of waterlogging, Figure S2: Protein content in sesame leaves from plants at vegetative stage under different waterlogging durations. Mean (±SD) was calculated from three replicates for each treatment.
