# Supplementary material for: Oxidative Damage and Antioxidant Defense in Sesamum indicum after Different Waterlogging Durations
Source: Plants (Basel). 2019 Jun 29;8(7):196. doi: 10.3390/plants8070196 (PMC6681296; doi:10.3390/plants8070196)
Supplement: Supplementary file 1 [file plants-08-00196-s001.pdf]

plants

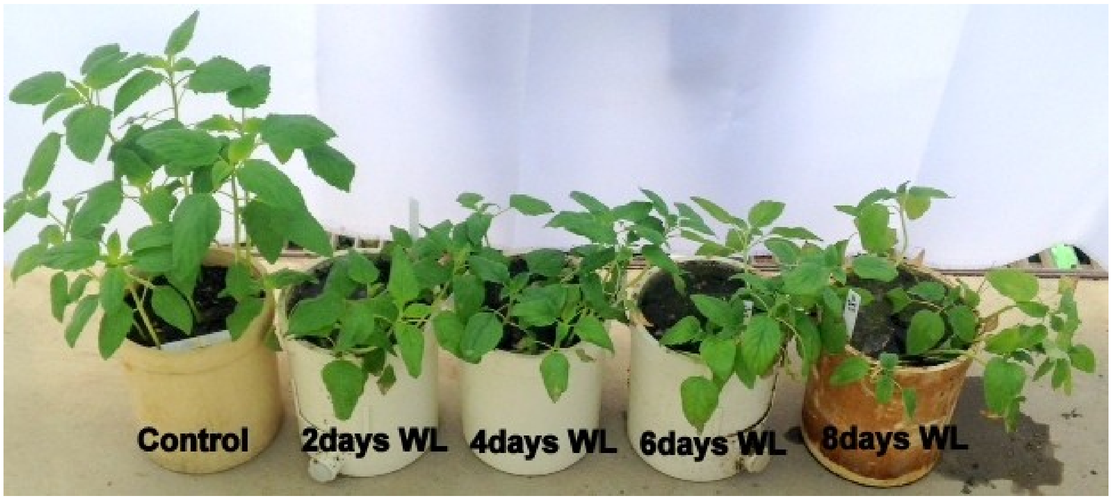

Supplementary Figure S1. Phenotypic appearance of sesame plants under different levels of waterlogging.

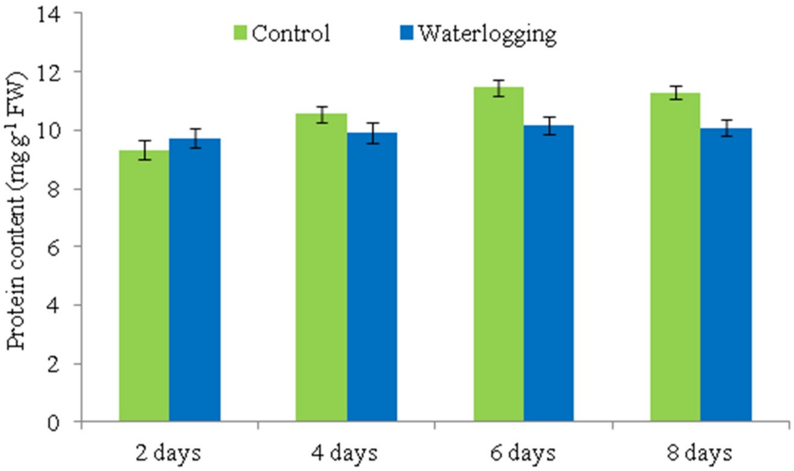

Supplementary Figure S2. Protein content in sesame leaves from plants at vegetative stage under different waterlogging durations. Mean (±SD) was calculated from three replicates for each treatment.
